# Supplementary material for: Observing and braiding topological Majorana modes on programmable quantum simulators
Source: Nat Commun. 2023 Apr 21;14:2286. doi: 10.1038/s41467-023-37725-0 (PMC10121601; doi:10.1038/s41467-023-37725-0)
Supplement: Supplementary file 1 — Supplementary Information [file 41467_2023_37725_MOESM1_ESM.pdf]

Supplementary Information for "Observing and braiding topological Majorana modes on programmable quantum simulators"

Nikhil Harle,[1,2] Oles Shtanko[3], and Ramis Movassagh[2]

[1] *Department of Physics, Yale University, New Haven CT, 06520, USA*
[2] *IBM Quantum, MIT-IBM Watson AI lab, Cambridge MA, 02142, USA*
[3] *IBM Quantum, IBM Research – Almaden, San Jose CA, 95120, USA*

SUPPLEMENTARY NOTE 1: EIGENMODES AND THEIR PROPERTIES

Analyzing the eigenmodes of a system can be an effective way to describe its dynamics. In this section, we introduce and analyze the properties of eigenmodes for time-periodic (Floquet) dynamics generated by the unitary operator U_F in Eq. (2). We prove three propositions concerning the eigenmodes of the system, which we then use in the following sections.

Proposition 1. ***(Existence of eigenmodes)*** *For any unitary U_F, there exists a complete set of eigenoperators (eigenmodes) Δ_b and real eigenfrequencies ω_b, $1 \le b \le 4^N$, such that*

$$\begin{aligned} U_F^\dagger \Delta_b U_F &= e^{-i\omega_b}\Delta_b, \\ \mathrm{Tr}\,(\Delta_b^\dagger \Delta_{b'}) &= 2^N \delta_{bb'}, \end{aligned} \tag{1}$$

where N is the number of qubits and $\delta_{bb'}$ is the Kronecker delta.

Proof. Consider $\mathcal{P} = \{P_\alpha : \alpha = 1,\dots,d\}$ as a complete set of $2^N \times 2^N$ Pauli basis operators, where $d = 4^N$ is the dimension of the space they span. The unitary transformation of each basis operator is given by

$$U_F^\dagger P_\alpha U_F = \sum_{\beta=1}^{d} E_{\alpha\beta} P_\beta, \tag{2}$$

where $E_{\alpha\beta} := 2^{-N}\mathrm{Tr}\,(U_F^\dagger P_\alpha U_F P_\beta)$ are matrix elements of a real orthogonal matrix,

$$E_{\alpha\beta} = E^*_{\alpha\beta}, \quad EE^T = E^T E = I. \tag{3}$$

To prove that E is orthogonal, we use Eq. (2) to express

$$U_F^\dagger P_\alpha P_{\alpha'} U_F = \sum_{\beta,\beta'=1}^{d} E_{\alpha\beta}E_{\alpha'\beta'} P_\beta P_{\beta'}. \tag{4}$$

Next, we take the trace for the two sides of Eq. (4) and use the orthogonality of Pauli matrices, $\mathrm{Tr}\,(P_\alpha P_\beta) = 2^N\delta_{\alpha\beta}$. Using the fact that $E_{\alpha\beta} = E^*_{\alpha\beta}$, we get that

$$\sum_{\beta=1}^{d} E_{\alpha\beta}E^T_{\beta\alpha'} = \sum_{\beta=1}^{d} E_{\alpha'\beta}E^T_{\beta\alpha} = \delta_{\alpha\alpha'}. \tag{5}$$

Since the matrix E is orthogonal, it has an orthonormal set of eigenstates and eigenvalues that lie on the unit circle in the complex plane:

$$v_b^T E = e^{-i\omega_b} v_b^T. \tag{6}$$

Using this set, we construct the operators

$$\Delta_b = \sum_{\alpha=1}^{d} v_{b\alpha} P_\alpha, \tag{7}$$

where $v_{b\alpha}$ stands for the α-th entry of v_b. These operators satisfy

$$\begin{aligned} U_F^\dagger \Delta_b U_F &= \sum_{\alpha=1}^{d} v_{b\alpha} U_F^\dagger P_\alpha U_F = \sum_{\alpha,\beta=1}^{d} v_{b\alpha}E_{\alpha\beta}P_\beta \\ &= e^{-i\omega_b}\sum_{\beta=1}^{d} v_{b\beta}P_\beta = e^{-i\omega_b}\Delta_b, \end{aligned} \tag{8}$$

which proves the first equation in Eq. (1). Furthermore, the orthogonality of the eigenstates of the operator E leads to the expression

$$\begin{aligned} \mathrm{Tr}\,(\Delta_b^\dagger \Delta_{b'}) &= \sum_{\alpha,\beta=1}^{d} v^*_{b\alpha}v_{b\beta}\mathrm{Tr}\,(P_\alpha P_\beta) \\ &= 2^N \sum_{\alpha=1}^{d} v^*_{b\alpha}v_{b'\alpha} = 2^N\delta_{bb'}, \end{aligned} \tag{9}$$

proving the second equation in Eq. (1) and thus completing our proof. □

The spectrum of the system can contain degeneracies. If we define B as the full set of eigenmodes, then $B_\omega = \{b_1,\dots,b_m\}$ is defined as the subset of eigenmodes with the same frequency $\omega_{b_k} = \omega$ for $1 \le k \le m$ in the limit $N \to \infty$. We define $\overline{B_\omega} = B\backslash B_\omega$ as the set of eigenmodes with frequencies different from ω.

Proposition 2. *For the dynamics in Eq. (2), non-trivial eigenmodes $\Delta_b \ne I$ and Majorana operators satisfy*

$$\Delta_b = \sum_{\mu=1}^{2N} w_{\mu b}\gamma_\mu + C_\perp, \quad \gamma_\mu = \sum_{b\in B} w^*_{\mu b}\Delta_b, \tag{10}$$

where $w_{\mu b}$ are complex-valued coefficients and $C_\perp$ is an operator that satisfies $\mathrm{Tr}\,(\gamma_\mu C_\perp) = 0$ for all μ.

Proof. First, we prove the second equation. Since Δ_b form a complete basis, we have

$$O = \frac{1}{2^N} \sum_{b \in B} \mathrm{Tr}\,(O\Delta_b^\dagger)\Delta_b. \tag{11}$$

Then, the second part of Eq. (10) follows from this expression by setting $w^*_{\mu b} := 2^{-N}\mathrm{Tr}\,(\gamma_\mu \Delta_b^\dagger)$. Any many-body operator can be written as a decomposition of Majorana operators,

$$\Delta_b = \sum_{\mu=1}^{2N} C^{(1)}_{b,\mu}\gamma_\mu + \sum_{\mu\nu=1}^{2N} C^{(2)}_{b,\mu\nu}\gamma_\mu\gamma_\nu + \dots\ . \tag{12}$$

Multiplying both sides by the operator γ_μ and taking the trace, we get

$$\mathrm{Tr}\,(\Delta_b \gamma_\mu) = 2^N C^{(1)}_{b,\mu}. \tag{13}$$

That is, we derive the expression

$$C^{(1)}_{b,\mu} = w_{\mu b}. \tag{14}$$

which leads to Eq. (10) and completes our proof. □

As a useful tool for analytical derivations, we introduce the Fourier channel

$$\mathcal{F}_\omega(\cdot) := \lim_{N,D\to\infty} \frac{1}{D} \sum_{n=0}^{D-1} e^{i\omega n} U_F^{\dagger n}(\cdot) U_F^n, \tag{15}$$

where we first take N and then D to infinity. The action of this channel is expressed in terms of the eigenmodes of the system by the following Proposition.

Proposition 3. *The action of the Fourier map in Eq.* (15) *can be expressed as*

$$\mathcal{F}_\omega(O) = \lim_{N\to\infty} \frac{1}{2^N} \sum_{b \in B_\omega} \mathrm{Tr}\,(O\Delta_b^\dagger)\Delta_b. \tag{16}$$

Proof. Using the decomposition in Eq. (11), we have

$$\mathcal{F}_\omega(O) = \lim_{N,D\to\infty} \frac{1}{D} \sum_{n=0}^{D-1} e^{i\omega n} \sum_{b\in B} \frac{1}{2^N} \mathrm{Tr}\,(O\Delta_b^\dagger) U_F^{\dagger n} \Delta_b U_F^n. \tag{17}$$

Now, using Proposition 1, we have

$$\mathcal{F}_\omega(O) = \lim_{N,D\to\infty} \frac{1}{2^N D} \sum_{n=0}^{D-1} \sum_{b\in B} e^{i(\omega-\omega_b)n} \mathrm{Tr}\,(O\Delta_b^\dagger)\Delta_b. \tag{18}$$

Using the property

$$\lim_{D\to\infty} \frac{1}{D} \sum_{n=0}^{D-1} e^{i\omega n} = \delta_{\omega,0}, \tag{19}$$

we get the expression

$$\mathcal{F}_\omega(O) = \lim_{N\to\infty} \frac{1}{2^N} \sum_{b\in B} \delta_{\omega-\omega_b,0} \mathrm{Tr}\,(O\Delta_b^\dagger)\Delta_b. \tag{20}$$

Next, using the definition of B_ω, we obtain the statement of this Proposition. □

If the system is non-interacting, i.e. $\lambda = 0$, the eigenmodes can be efficiently expressed in the free fermionic representation. The action of the Floquet unitary on the Majorana operators is then the linear map

$$U_F^\dagger \gamma_\mu U_F = \sum_{\nu=1}^{2N} u_{\mu\nu}\gamma_\nu, \tag{21}$$

where u is a $2N \times 2N$ unitary matrix

$$u = \exp(4\theta h_{xx}) \exp(4\phi h_z), \tag{22}$$

and h_z and h_{xx} are

$$\begin{aligned} h_z &= -\frac{1}{2} \sum_{k=0}^{N-1} \Big(|2k+1\rangle\langle 2k+2| - \text{h.c.} \Big), \\ h_{xx} &= \frac{1}{2} \sum_{k=0}^{N-2} \Big(|2k+2\rangle\langle 2k+3| - \text{h.c.} \Big). \end{aligned} \tag{23}$$

Using the solution of the eigenproblem

$$\psi_k^T u = e^{-i\omega_k^0} \psi_k^T, \tag{24}$$

we define the set of single-fermion modes

$$\Delta_k^0 = \sum_{\mu=1}^{2N} \psi_{k\mu}\gamma_\mu, \quad \{\Delta_k^{0\dagger}, \Delta_{k'}^0\} = 2\delta_{kk'}. \tag{25}$$

Since the unitary matrix in Eq. (22) is real-valued, the complex conjugate for both sides in Eq. (24) gives us another, opposite-frequency solution

$$(\psi_k^*)^T u = e^{i\omega_k^0} (\psi_k^*)^T. \tag{26}$$

This conclusion proves that the spectrum of the problem is zero-symmetric, i.e. for each mode k there exists an orthogonal mode $\sigma(k)$ such that $\psi_{\sigma(k)} = \psi_k^*$ and $\omega^0_{\sigma(k)} = -\omega_k^0$. This mathematical property is a reflection of the physical particle-hole symmetry.

For the non-interacting case, each many-body eigenmode in Eq. (1) and its frequency can be expressed as

$$\Delta_b = \Delta^0_{m_1} \dots \Delta^0_{m_K}, \qquad \omega_b = \sum_{i=1}^{K} \omega^0_{m_i}. \tag{27}$$

Each mode appears only once in the product, i.e. $m_i \neq m_j$, and the number of single-particle modes involved is $K \in \{1, \dots, 2N\}$. A crucial advantage of the free fermion representation is its efficiency in evaluating the eigenmodes on a classical computer by diagonalizing the unitary operator in Eq. (22).

Finally, we define the sets of approximate modes.

Definition 1. *An orthonormal operator set $\{\Delta_b'\}$ is called an ϵ-approximate set of eigenmodes if it satisfies*

$$U_F^\dagger \Delta_b' U_F = e^{-i\omega_b}\Delta_b' + \delta O, \quad \|\delta O\| \leq \epsilon, \tag{28}$$

where $\|\cdot\|$ is the operator norm.

Approximate modes behave similarly to regular eigenmodes up to times $\tau \propto 1/\epsilon$.

Let us consider the realistic case $D < \infty$ and in the presence of noise. In the experiment, we implement the map

$$\tilde{\mathcal{F}}_{D,\omega}(\cdot) := \frac{1}{D}\sum_{m=0}^{D-1} e^{i\omega n}\mathcal{E}_F^n(\cdot), \tag{29}$$

where $\mathcal{E}_F$ is a general map that includes unitary evolution and the effect of noise. The effect of this map can be approximated by

$$\mathcal{E}_F(\Delta_b) \approx e^{i\omega_b - \Gamma_b}\Delta_b \tag{30}$$

where Γ_b is the decay rate that includes both the effect of the noise and the finite lifetime of the approximate integral of motion Δ_b. This effect is visible in Fig. 5, especially in the presence of ZZ-gates. Then, the effect of noise and finite depth can be understood as

$$\tilde{\mathcal{F}}_{D,\omega}(O) \approx \sum_{b\in B} f_D(\omega - \omega_b, \Gamma_b)\mathrm{Tr}\,(O\Delta_b^\dagger)\Delta_b. \tag{31}$$

where the coefficients are

$$f_D(\omega, \Gamma) = \frac{1}{D}\frac{1 - e^{i\omega D - \Gamma D}}{1 - e^{i\omega - \Gamma}}. \tag{32}$$

This function generates the peak broadening and effective attenuation factor as $|f_D(\omega, \Gamma)| \leq 1$.

SUPPLEMENTARY NOTE 2: MAJORANA EIGENMODES

The topological Majorana modes Γ_s, $s \in \{L, R\}$, satisfy

$$U_F^\dagger \Gamma_s U_F = e^{-i\omega_M}\Gamma_s + O(1/\tau), \tag{33}$$

where ω_M are the Majorana mode frequencies, $\omega_M \in \{0, \pi\}$ in the limit $N \to \infty$, and τ is the lifetime. The Majorana modes are commonly described as "strong" if $\tau \to \infty$, while the case $\tau < \infty$ corresponds to "weak" modes.

For the topological Majorana modes, both strong and weak, we use the following form

$$\Gamma_s = \sum_{\mu=1}^{2N} \psi_\mu^s \gamma_\mu, \tag{34}$$

where γ_μ are physical Majorana operators and ψ_μ^s are real Majorana wave functions. In the non-interacting topological phase, $\lambda = 0$, there always exists a pair of solutions for the unitary in Eq. (22) which satisfies

$$\sum_{\mu=1}^{2N} u_{\mu\nu}\psi_\mu^s = \pm\psi_\nu^s \tag{35}$$

corresponding to MZMs (plus sign) and MPMs (minus sign). Therefore, non-interacting regimes are always characterized by *strong* Majorana modes. On the other hand, in the interacting regime, i.e. $|\lambda| > 0$, the existence of such operators is not guaranteed. However, for certain values of the angle (θ, ϕ) such modes are weak eigenmodes characterized by large $\tau \propto \exp(c/\lambda) \gg 1$ [1]. Even in the ideal case where we ignore the noise in the actual experiment, as will become clear in Eq. (39), the lifetime τ limits the maximum depth D such that $D/\tau \to 0$. In practice, this connection means that the Fourier components evaluated in Eq. (4) must be compared with the experimental results obtained for $\tau \gg D \gg 1$.

For both strong and weak modes, the wavefunctions ψ_μ^s must be properly normalized, as follows from the condition $\Gamma_s^2 = 1$. Indeed,

$$\Gamma_s^2 = \sum_{\nu,\nu'=1}^{2N} \psi_\nu^s\psi_{\nu'}^s\gamma_\nu\gamma_{\nu'} = \sum_{\nu=1}^{2N}(\psi_\nu^s)^2 = 1, \tag{36}$$

where we used the statistics of the Majorana operators, $\{\gamma_\mu, \gamma_\nu\} = 2\delta_{\mu\nu}$.

Next, we analyze how to detect Majorana modes using the Fourier components in Eq. (4) generated from the experiment. To do this, we express these components using the Fourier map in Eq. (15) as

$$\begin{aligned} F_\mu^s(0) &:= \lim_{N,D\to\infty}\frac{1}{D}\sum_{n=0}^{D-1}\langle\psi_0|U_F^{\dagger n}\gamma_\mu U_F^n|\psi_0\rangle \\ &= \langle\psi_0|\mathcal{F}_0(\gamma_\mu)|\psi_0\rangle. \end{aligned} \tag{37}$$

In the following section we will show how to distinguish true MZM modes from other trivial edge oscillations. For now, let us focus on zero-frequency MZMs and assume that they are the only pair of zero-frequency modes that have a non-zero overlap with the Majorana operators γ_μ. Then, from Proposition 3, this action is given by

$$\begin{aligned} \mathcal{F}_0(\gamma_\mu) = &\sum_{s\in\{L,R\}}\psi_\mu^s \lim_{N,D\to\infty}\frac{1}{D}\sum_{n=0}^{D-1} U_F^{\dagger n}\Gamma_s U_F^n \\ &+ \lim_{N,D\to\infty}\frac{1}{D}\sum_{n=0}^{D-1}\frac{1}{2^N}\sum_{b\in\overline{B_0}} w_{b\mu}^* e^{-i\omega_b n}\Delta_b. \end{aligned} \tag{38}$$

where $\overline{B_0}$ is the complementary to zero frequency subspace B_0. Under the limit over D the last term of this

expression vanishes. At the same time, the first term can be simplified with Eq. (33) as

$$\lim_{N,D\to\infty}\frac{1}{D}\sum_{n=0}^{D-1}U_F^{\dagger n}\Gamma_s U_F^n=\lim_{N,D\to\infty}\frac{1}{D}\sum_{n=0}^{D-1}(\Gamma_s+O(n/\tau)) \tag{39}$$

Assuming that $\lim_{D\to\infty} D/\tau = 0$, we get

$$\mathcal{F}_0(\gamma_\mu)=\lim_{N\to\infty}\sum_{s\in\{L,R\}}\psi_\mu^s\Gamma_s\equiv\lim_{N\to\infty}(\psi_\mu^L\Gamma_L+\psi_\mu^R\Gamma_R), \tag{40}$$

Using Eqs. (40) and (34), we transform Eq. (37) into

$$F_\mu^\alpha(0)=\lim_{N\to\infty}\sum_{s\in\{L,R\}}\sum_{\mu'=1}^{2N}\psi_\mu^s\psi_{\mu'}^s\langle\psi_0|\gamma_{\mu'}^\alpha|\psi_0\rangle. \tag{41}$$

where we restored the Pauli representation index for Majorana operators. Using $\langle\psi_0|\gamma_\mu^L|\psi_0\rangle = \delta_{\mu,1}$ and $\langle\psi_0|\gamma_\mu^R|\psi_0\rangle = \delta_{\mu,2N}$, we rewrite

$$\begin{aligned}F_\mu^L(0)&=\lim_{N\to\infty}(\psi_\mu^L\psi_1^L+\psi_\mu^R\psi_1^R),\\ F_\mu^R(0)&=\lim_{N\to\infty}(\psi_\mu^L\psi_{2N}^L+\psi_\mu^R\psi_{2N}^R).\end{aligned} \tag{42}$$

Taking into account that the eigenmodes are exponentially suppressed away from the respective boundaries of the system, i.e. $\psi_1^R \sim \psi_{2N}^L \sim 2^{-\Theta(N)}$, we obtain the expressions

$$\begin{aligned}F_\mu^L(0)&=\lim_{N\to\infty}\psi_\mu^L\psi_1^L,\\ F_\mu^R(0)&=\lim_{N\to\infty}\psi_\mu^R\psi_{2N}^R,\end{aligned} \tag{43}$$

from which we derive

$$\begin{aligned}\psi_\mu^L&=F_\mu^L(0)/\sqrt{F_1^L(0)},\\ \psi_\mu^R&=F_\mu^R(0)/\sqrt{F_{2N}^R(0)}.\end{aligned} \tag{44}$$

The evaluation can be done in a similar way for MPM. The final expression is the same as Eq. (3) in the main text.

In the case of finite circuit depth and in the presence of noise, we use Eq. (31) to modify Eq. (44) as

$$\psi_\mu^{L,R}\to\sqrt{|f(\omega,\Gamma)|}\,\psi_\mu^{L,R}, \tag{45}$$

where $\omega \sim 2^{-O(N)}$ is the frequency of the Majorana mode for a system of finite size and Γ is the effective decay rate. Since $|f(\omega,\Gamma)| \leq 1$, the approximation given by Eq. (30) leads to a uniform damping of the wavefunction as seen in Eq. (45). Normalizing the wavefunction eliminates this effect. However, more general noise models would result in more complex effects.

SUPPLEMENTARY NOTE 3: TWO-POINT CORRELATION FUNCTION

In this section we will assume a non-interacting scenario where $\lambda = 0$. For simplicity, we will focus on zero-frequency modes, although π-frequency modes can be treated similarly. First, we will establish a connection between the two-point function in Eq. (5) and an expectation of the Fourier map given by

$$T_{\mu\nu}=\langle\tilde{\psi_0}|\mathcal{F}_0(\gamma_\mu\gamma_\nu)|\tilde{\psi_0}\rangle, \tag{46}$$

where we have chosen the initial product state in the form of the product state $|\tilde{\psi_0}\rangle = |\psi_a\rangle|s_2\rangle\ldots|s_{N-1}\rangle|\psi_a\rangle$, where $|\psi_a\rangle = \cos a|0\rangle + i\sin a|1\rangle$, and $|s_i\rangle$ are arbitrary states in the Z basis, $s_i \in \{0,1\}$. Next, we express the Majorana operators using free fermion eigenmodes as

$$\gamma_\mu=\sum_{k=1}^{2N}\psi_{k\mu}^*\Delta_k^0, \tag{47}$$

where the wavefunctions $\psi_{k\mu}$ are defined in Eq. (24). This decomposition allows us to write

$$\begin{aligned}&\mathcal{F}_0(\gamma_\mu\gamma_\nu)=\\ &\quad=\lim_{N,D\to\infty}\frac{1}{D}\sum_{n=0}^{D-1}\sum_{k,k'=1}^{2N}\psi_{k\mu}^*\psi_{k'\nu}^*e^{-i(\omega_k^0+\omega_{k'}^0)n}\Delta_k^0\Delta_{k'}^0\\ &\quad=\lim_{N\to\infty}(A_{\mu\nu}+B_{\mu\nu}),\end{aligned} \tag{48}$$

where the variable $A_{\mu\nu}$ denotes the contribution from the set of zero-frequency modes (B_0), while $B_{\mu\nu}$ represents the contribution from pairs of modes with opposite frequencies from the complementary set $\overline{B_0}$,

$$\begin{aligned}A_{\mu\nu}&=\sum_{k,k'\in B_0}\psi_{k\mu}\psi_{k'\nu}\Delta_k^0\Delta_{k'}^0,\\ B_{\mu\nu}&=\sum_{k\in\overline{B_0}}\psi_{k\mu}^*\psi_{\sigma(k)\nu}^*\Delta_k^0\Delta_{\sigma(k)}^0\\ &=\sum_{k\in\overline{B_0}}\psi_{k\mu}^*\psi_{k\nu}\Delta_k^0\Delta_k^{0\dagger}\end{aligned} \tag{49}$$

The remaining terms vanish in the limit $D \to \infty$. Here $\sigma(k)$ represents the opposite frequency mode with respect to mode k. We also take advantage of the fact that we can always choose the zero-frequency modes $k \in B_0$ to be real-valued, $\psi_{k\mu} = \psi_{k\mu}^*$, while the remaining modes satisfy the relations $\psi_{\sigma(k)\mu} = \psi_{k\mu}^*$, $\omega_{\sigma(k)}^0 = -\omega_k^0$, and $\Delta_{\sigma(k)}^0 = \Delta_k^{0\dagger}$.

Using these notations, the target two-point correlation function can be expressed as

$$T_{\mu\nu}=\lim_{N\to\infty}\Big(\langle\tilde{\psi_0}|A_{\mu\nu}|\tilde{\psi_0}\rangle+\langle\tilde{\psi_0}|B_{\mu\nu}|\tilde{\psi_0}\rangle\Big). \tag{50}$$

Next, we use Eq. (10) to express the expectation value

$$\langle\tilde{\psi}_0|\Delta_k^0\Delta_{k'}^0|\tilde{\psi}_0\rangle = \sum_{\mu,\mu'=1}^{2N} \psi_{k\mu}\psi_{k'\mu'}\langle\tilde{\psi}_0|\gamma_\mu\gamma_{\mu'}|\tilde{\psi}_0\rangle,$$
$$\langle\tilde{\psi}_0|\Delta_k^0\Delta_{k'}^{0\dagger}|\tilde{\psi}_0\rangle = \sum_{\mu,\mu'=1}^{2N} \psi_{k\mu}\psi^*_{k'\mu'}\langle\tilde{\psi}_0|\gamma_\mu\gamma_{\mu'}|\tilde{\psi}_0\rangle. \tag{51}$$

The expected values on the right-hand side of these equations can be expressed in terms of the chosen product state $|\tilde{\psi}_0\rangle$ as

$$\begin{aligned}\langle\tilde{\psi}_0|\gamma_\mu\gamma_{\mu'}|\tilde{\psi}_0\rangle = \delta_{\mu\mu'} &+ iC_1(\delta_{\mu 1}\delta_{\mu' 2N} - \delta_{\mu 2N}\delta_{\mu' 1}) \\ &+ iC_2(\delta_{\mu 1}\delta_{\mu' 2} - \delta_{\mu 2}\delta_{\mu' 1}) \\ &+ iC_2(\delta_{\mu 2N-1}\delta_{\mu' 2N} - \delta_{\mu 2N}\delta_{\mu' 2N-1}),\end{aligned} \tag{52}$$

where we used the notations

$$\begin{aligned}C_1 &= \langle\psi_a|Y|\psi_a\rangle^2\prod_{i=2}^{N-1}\langle s_i|(-Z_i)|s_i\rangle = (-1)^{N+S}\sin^2(2a),\\ C_2 &= \langle\psi_a|Z|\psi_a\rangle = \cos 2a.\end{aligned} \tag{53}$$

where $S = \sum_{i=2}^{N-1} s_i$. We use these expressions to obtain

$$\begin{aligned}\langle\tilde{\psi}_0|\Delta_k^0\Delta_{k'}^0|\tilde{\psi}_0\rangle = \sum_{\mu=1}^{2N}\psi_{k\mu}\psi_{k'\mu} &+ iC_1(\psi_{k1}\psi_{k'2N} - \psi_{k2N}\psi_{k'1}) \\ &+ iC_2(\psi_{k1}\psi_{k'2} - \psi_{k1}\psi_{k'2} \\ &\quad + \psi_{k2N-1}\psi_{k'2N} - \psi_{k2N}\psi_{k'2N-1}).\end{aligned} \tag{54}$$

and

$$\begin{aligned}\langle\tilde{\psi}_0|\Delta_k^0\Delta_{k'}^{0\dagger}|\tilde{\psi}_0\rangle = \sum_{\mu=1}^{2N}\psi_{k\mu}\psi^*_{k'\mu} &+ iC_1(\psi_{k1}\psi^*_{k'2N} - \psi_{k2N}\psi^*_{k'1}) \\ &+ iC_2(\psi_{k1}\psi^*_{k'2} - \psi_{k1}\psi^*_{k'2} \\ &\quad + \psi_{k2N-1}\psi^*_{k'2N} - \psi_{k2N}\psi^*_{k'2N-1}).\end{aligned} \tag{55}$$

For real-valued zero frequency modes, we use the orthogonality condition

$$\forall k \in B_0: \qquad \sum_{\mu=1}^{2N}\psi_{k\mu}\psi_{k'\mu} = \delta_{kk'}. \tag{56}$$

As a result, the expression for the contribution of the zero frequency modes to the two-point correlation function can be written as

$$\begin{aligned}\langle\tilde{\psi}_0|A_{\mu\nu}|\tilde{\psi}_0\rangle &= \sum_{k,k'\in B_0}\psi_{k\mu}\psi_{k'\nu}\langle\tilde{\psi}_0|\Delta_k^0\Delta_{k'}^0|\tilde{\psi}_0\rangle \\ &= \sum_{k\in B_0}\psi_{k\mu}\psi_{k\nu} \\ &\quad + iC_1\Big(\sum_{k\in B_0}\psi_{k\mu}\psi_{k1}\sum_{k'\in B_0}\psi_{k'\nu}\psi_{k'2N} - (\mu\leftrightarrow\nu)\Big) \\ &\quad + iC_2\Big(\sum_{k\in B_0}\psi_{k\mu}\psi_{k1}\sum_{k'\in B_0}\psi_{k'\nu}\psi_{k'2} - (\mu\leftrightarrow\nu)\Big) \\ &\quad + iC_2\Big(\sum_{k\in B_0}\psi_{k\mu}\psi_{k2N-1}\sum_{k'\in B_0}\psi_{k'\nu}\psi_{k'2N} - (\mu\leftrightarrow\nu)\Big),\end{aligned} \tag{57}$$

where $(\mu\leftrightarrow\nu)$ is the same term as before with the indices μ and ν swapped.

Similarly, the second part has the form

$$\begin{aligned}\langle\tilde{\psi}_0|B_{\mu\nu}|\tilde{\psi}_0\rangle &= \sum_{k\in\overline{B_0}}\psi^*_{k\mu}\psi_{k\nu} \\ &\quad + \Big(iC_1\sum_{k\in\overline{B_0}}\psi^*_{k\mu}\psi_{k1}\psi_{k\nu}\psi^*_{k2N} \\ &\quad + iC_2\sum_{k\in\overline{B_0}}\psi^*_{k\mu}\psi_{k1}\psi_{k\nu}\psi^*_{k2} \\ &\quad + iC_2\sum_{k\in\overline{B_0}}\psi^*_{k\mu}\psi_{k2N-1}\psi_{k\nu}\psi^*_{k2N} - (\mu\leftrightarrow\nu)\Big).\end{aligned} \tag{58}$$

Since we assume that the bulk of the system is delocalized, the wavefunctions of single-fermion modes with non-zero frequency must satisfy $\psi_{k\mu}\propto O(N^{-1/2})$. This means that all terms in Eq. (58) except the first have $O(N^{-1})$ scaling and therefore vanish in the limit $N\to\infty$. Therefore, combining the contributions in Eqs. (57) and (58) we get

$$\begin{aligned}T_{\mu,\nu} = \delta_{\mu\nu} + \lim_{N\to\infty}\Big[&iC_1\sum_{k\in B_0}\psi_{k\mu}\psi_{k1}\sum_{k'\in B_0}\psi_{k'\nu}\psi_{k'2N} \\ &+ iC_2\sum_{k\in B_0}\psi_{k\mu}\psi_{k1}\sum_{k'\in B_0}\psi_{k'\nu}\psi_{k'2} \\ &+ iC_2\sum_{k\in B_0}\psi_{k\mu}\psi_{k2N-1}\sum_{k'\in B_0}\psi_{k'\nu}\psi_{k'2N} \\ &- (\mu\leftrightarrow\nu)\Big].\end{aligned} \tag{59}$$

Next, we analyze the behavior of this function for a few characteristic values of μ and ν. First we consider the combination $\mu = 1$ and $\nu = 2$ and assume that the wavefunctions $\psi_{k\mu}$ for zero modes $k\in B_0$ are strongly localized, i.e. $\psi_{k\mu}\psi_{k\nu}\le 2^{-c|\mu-\nu|}$, $c>0$. Then the correlation function takes the form

$$T_{1,2} = iC_2\sum_{k\neq k'\in B_0}(\psi_{k1}^2\psi_{k'2}^2 - \psi_{k2}^2\psi_{k'1}^2). \tag{60}$$

In particular, if the only modes are Majorana modes, then $\psi_1^L \psi_2^R \sim \psi_1^L \psi_2^R \sim O(2^{-\Theta(N)})$, and this quantity vanishes in the thermodynamic limit. In contrast, if there is a localized state at the boundary of the system, there exists a pair $b \neq b'$ for which $T_{1,2} = O(1)$.

At the same time, the correlation function for $\mu = 1$ and $\nu = 2N$ has the form

$$T_{1,2N} = iC_1 \sum_{b,b' \in B_0} (\psi_{k1}^2 \psi_{k'2N}^2 - \psi_{k2N}^2 \psi_{k'1}^2). \quad (61)$$

This expression does not vanish for both Majorana and trivial modes. For other points μ and ν far from the boundaries, the two-point correlation function vanishes.

To model the trivial system with approximate zero-energy localized boundary eigenmodes, we consider the Hamiltonian

$$H(t) = \sum_{j=1}^{N-1} J_j(t)\, X_j X_{j+1} + \sum_{j=1}^{N} h_j(t) Z_j, \quad (62)$$

with the same protocol. The couplings and fields for the bulk qubits are the same, $J_j T = \pi/16$ for $j \neq 1, N-1$ and $h_j T = \pi/4$ for $j \neq 1, N$. At the same time, we set $J_1 = J_{N-1} = h_1 = h_N = 0$. Due to decoupling of the boundary qubits, Majorana operators corresponding to these qubits (i.e. γ_1, γ_2, γ_{2N-1}, and γ_{2N}) are integrals of motion.

SUPPLEMENTARY NOTE 4: BRAIDING MAP

In this section, we provide a rigorous proof of the properties of the braiding map in Eq. (7) in the main text. First, we formulate Lemma 1, which establishes the action of the map on MZM operators.

Lemma 1. *Suppose Eq.* (40) *holds and* $\psi_1^L = \psi_{2N}^R = \xi$, $\xi^2 \geq 1/2$. *Then for the angle* $\alpha_0 = \arcsin(1/\sqrt{2}\xi)$ *the action of the map in Eq.* (7) *on MZM operators is*

$$\mathcal{E}_{\alpha_0}(\Gamma_R) = p\Gamma_L, \quad \mathcal{E}_{\alpha_0}(\Gamma_L) = -p\Gamma_R, \quad (63)$$

where $p = \sqrt{2\xi^2 - 1}$.

Lemma 1 leads to Eq. (8) in the main text. This lemma applies only to systems where MZMs are the only zero-frequency modes overlapping with single-fermion operators, as manifested by Eq. (40). At the same time, it applies to a generic setting including the interacting case $|\lambda| > 0$.

Next, we formulate Lemma 2, which gives the expression for the map action on Majorana operators.

Lemma 2. *Under conditions of Lemma 1, the map in Eq.* (7) *satisfies*

$$\mathcal{E}_{\alpha_0}(\gamma_\mu) = p(\psi_\mu^R \Gamma_L - \psi_\mu^L \Gamma_R) + \delta C, \quad (64)$$

where the norm of the correction operator is bounded as

$$\begin{aligned} \|\delta C\| &\leq \frac{1}{\xi^2} \max_\mu \sqrt{\sum_\nu \kappa_{\mu\nu}^2}, \\ \kappa_{\mu\nu} &= \lim_{N\to\infty} \sum_{k \in \overline{B_0}} \psi_{k\mu}^* \psi_{k\nu}^* (\psi_{k1}^2 + \psi_{k2N}^2), \end{aligned} \quad (65)$$

where $\|\cdot\|$ *is the spectral norm.*

This conclusion leads us to Eq. (9) when the bulk modes are delocalized. Indeed, in this case $\kappa_{\mu\nu} = O(N^{-1})$, therefore the absolute value in Eq. (65) scales as $O(N^{-1/2})$ and the correction vanishes in the thermodynamic limit $N \to \infty$.

As an alternative to the theoretical prediction in Lemma 1, we explore the possibility of finding the correct angle α_0 by optimization. To illustrate this method, we run the circuit for multiple angles and find the optimal value of α_0. To estimate the braiding quality, we propose a cost function that favors a braided wavefunction if it is located at the opposite boundary of the chain. In particular, for braiding the left eigenmode, our cost function is given by

$$\mathcal{L}(\alpha_0) = \sum_{x=1}^{N} \Big(|\tilde{\psi}_{2x-1}^L|^2 + |\tilde{\psi}_{2x}^L|^2 \Big) (N-x)^2, \quad (66)$$

where $\tilde{\psi}_\mu^{L,R}$ are the braided wavefunctions in Eq. (10) for the angle α_0. The cost functions for different runs on the same device are shown in Fig. 1 with the same parameters as in Fig. (3). We use a simple polynomial approximation to find the optimal angle α_0 corresponding to the minimum of the approximation of the curve. In our experiment, the optimal value (0.256934π) differs slightly from the theoretical value (0.263127π). This difference is due to the presence of disorder and noise, which modify the original Hamiltonian dynamics.

Below we provide the proofs for both Lemma 1 and Lemma 2.

Proof of Lemma 1. As a first step, we use the commutativity $[U_F, \Gamma_s] = 0$ for MZM operators and the decomposition in Eq. (34) to rewrite

$$\begin{aligned} \mathcal{E}_\alpha(\Gamma_s) &= \lim_{N,D\to\infty} \frac{1}{D} \sum_{n=0}^{D-1} U_F^{\dagger n} V^\dagger(\alpha) \Gamma_s V(\alpha) U_F^n \\ &= \lim_{N,D\to\infty} \frac{1}{D} \sum_{n=0}^{D-1} \sum_{\mu=1}^{2N} \psi_\mu^s U_F^{\dagger n} V^\dagger(\alpha) \gamma_\mu V(\alpha) U_F^n. \end{aligned} \quad (67)$$

where we define $V(\alpha) := \exp(-\alpha\gamma_1\gamma_{2N})$. Now we can use the decomposition $V(\alpha) = \cos\alpha\, I - \sin\alpha\, \gamma_1\gamma_{2N}$ and commutation relation between Majorana operators to ex-

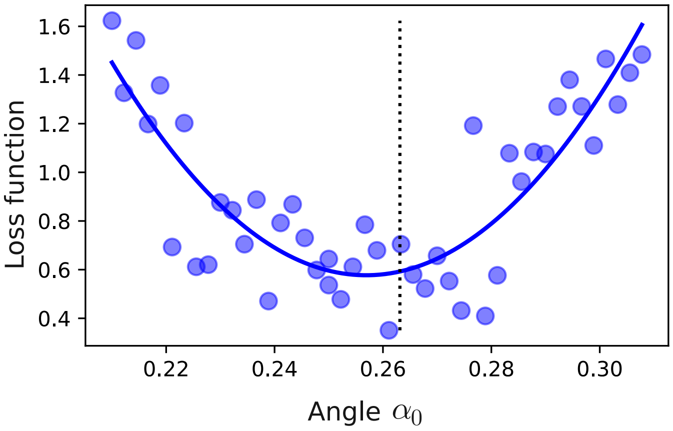

Supplementary Figure 1. **Finding the angle by optimization**. Cost function in Eq. (66) derived from experimental data (dots) approximated by a polynomial $I(\alpha_0) = a_1\alpha_0^2 + a_2\alpha_0 + a_3$ (solid curve), where a_i are coefficients. The dotted vertical line shows the theoretically predicted value of the angle α_0. We use a 5-qubit system on the *ibm_hanoi* device with parameters $\phi = \pi/16$, $\theta = \pi/4$, and $\varphi = 0$ and maximum number of cycles $D = 11$.

press

$$V^\dagger(\alpha)\gamma_\mu V(\alpha) = \gamma_\mu - \delta_{\mu 1}(2\sin^2\alpha\gamma_1 + \sin 2\alpha\gamma_{2N}) - \delta_{\mu 2N}(2\sin^2\alpha\gamma_{2N} - \sin 2\alpha\gamma_1). \tag{68}$$

Combining this result with Eq. (67), we get

$$\mathcal{E}_\alpha(\Gamma_s) = \Gamma_s - (2\psi_1^s\sin^2\alpha - \psi_{2N}^s\sin 2\alpha)\mathcal{F}_0(\gamma_1) - (2\psi_{2N}^s\sin^2\alpha + \psi_1^s\sin 2\alpha)\mathcal{F}_0(\gamma_{2N}), \tag{69}$$

where we use the notation for the Fourier map from Eq. (15). Then, using the property in Eq. (40), we get the expression

$$\mathcal{E}_\alpha(\Gamma_s) = \Gamma_s - \lim_{N\to\infty}\psi_1^L(2\psi_1^s\sin^2\alpha - \psi_{2N}^s\sin 2\alpha)\Gamma_L - \lim_{N\to\infty}\psi_{2N}^R(2\psi_{2N}^s\sin^2\alpha + \psi_1^s\sin 2\alpha)\Gamma_R. \tag{70}$$

Inserting the particular values $s \in \{L, R\}$ and taking into account that $\psi_{2N}^L \sim \psi_1^R \sim O(2^{-\Theta(N)})$ vanish as $N \to 0$, we get

$$\mathcal{E}_\alpha(\Gamma_R) = (1 - 2(\psi_{2N}^R)^2\sin^2\alpha)\Gamma_R + \sin 2\alpha\,\psi_1^L\psi_{2N}^R\Gamma_L,$$
$$\mathcal{E}_\alpha(\Gamma_L) = (1 - 2(\psi_1^L)^2\sin^2\alpha)\Gamma_L - \sin 2\alpha\,\psi_1^L\psi_{2N}^R\Gamma_R. \tag{71}$$

By putting $\psi_1^L = \psi_{2N}^R = \xi$, according to the Lemma's conditions, and choosing the angle

$$\alpha \to \alpha_0 = \arcsin\frac{1}{\sqrt{2}\xi}, \tag{72}$$

expression in Eq. (71) converts into the statement of the Lemma. This step concludes our proof.

Proof of Lemma 2. Assuming that Majorana modes are the only single-fermion modes with zero frequency, we can write

$$\gamma_\mu = \psi_\mu^L\Gamma_L + \psi_\mu^R\Gamma_R + \sum_{k\in\overline{B_0}}\psi_{k\mu}^*\Delta_k^0, \tag{73}$$

where $\overline{B_0}$ is the set of single-fermion modes in Eq. (25) whose frequencies are distinct from zero. Then, the action of the target map on Majorana operator is

$$\mathcal{E}_{\alpha_0}(\gamma_\mu) = \psi_\mu^L\mathcal{E}_{\alpha_0}(\Gamma_L) + \psi_\mu^R\mathcal{E}_{\alpha_0}(\Gamma_R) + \sum_{k\in\overline{B_0}}\psi_{k\mu}^*\mathcal{E}_{\alpha_0}(\Delta_k^0) = p(\psi_\mu^R\Gamma_L - \psi_\mu^L\Gamma_R) + \sum_{k\in\overline{B_0}}\psi_{k\mu}^*\mathcal{E}_{\alpha_0}(\Delta_k^0), \tag{74}$$

where we used Lemma 1 to express the action of the target map on topological Majorana operators $\Gamma_{L,R}$. The last term, in turn, can be evaluated using the explicit form of the map and Eq. (25),

$$\mathcal{E}_\alpha(\Delta_k^0) = \lim_{N,D\to\infty}\frac{1}{D}\sum_{n=0}^{D-1}e^{-i\omega_k^0 n}U_F^{\dagger n}V^\dagger(\alpha)\Delta_k^0 V(\alpha)U_F^n = \lim_{N,D\to\infty}\frac{1}{D}\sum_{n=0}^{D-1}\sum_{\nu=1}^{2N}\psi_{k\nu}e^{-i\omega_k^0 n}U_F^{\dagger n}V^\dagger(\alpha)\gamma_\nu V(\alpha)U_F^n. \tag{75}$$

Using the result in Eq. (68), we get

$$\mathcal{E}_\alpha(\Delta_k^0) = \lim_{N,D\to\infty}\frac{1}{D}\sum_{n=0}^{D-1}e^{-2i\omega_k^0 n}\Delta_k^0 - (2\psi_{k1}\sin^2\alpha - \psi_{k2N}\sin 2\alpha)\mathcal{F}_{-\omega_k^0}(\gamma_1) - (2\psi_{k2N}\sin^2\alpha + \psi_{k1}\sin 2\alpha)\mathcal{F}_{-\omega_k^0}(\gamma_{2N}), \tag{76}$$

where we use the notation for the Fourier map from Eq. (4). The first term of this expression vanishes for the set $k \in \overline{B_0}$. In turn, the action of the Fourier map is

$$\mathcal{F}_{-\omega_k}(\gamma_\mu) = \sum_{k'\in\overline{B_0}}\psi_{k'\mu}^*\Delta_{k'}^0\lim_{N,D\to\infty}\frac{1}{D}\sum_{n=0}^{D-1}e^{-i(\omega_k^0+\omega_{k'}^0)n} = \psi_{k\mu}\Delta_k^{0\dagger}, \tag{77}$$

where we used the fact that the wave-functions corresponding to opposite frequencies, i.e. $\omega_{k'}^0 = -\omega_k^0$, satisfy $\psi_{k'\mu} = \psi_{k\mu}^*$ and $\Delta_{k'}^0 = \Delta_k^{0\dagger}$. Then, Eq. (76) takes the form

$$\mathcal{E}_{\alpha_0}(\Delta_k^0) = -2\sin^2\alpha_0(\psi_{k1}^2 + \psi_{k2N}^2)\Delta_k^{0\dagger} = -\frac{1}{\xi^2}(\psi_{k1}^2 + \psi_{k2N}^2)\Delta_k^{0\dagger}. \tag{78}$$

Inserting this expression into Eq. (74), we finally get

$$\begin{aligned}\mathcal{E}_{\alpha_0}(\gamma_\mu) &= p(\psi_\mu^R\Gamma_L - \psi_\mu^L\Gamma_R)\\ &\quad -\frac{1}{\xi^2}\sum_{k\in\overline{B_0}}\psi_{k\mu}^*(\psi_{k1}^2+\psi_{k2N}^2)\Delta_k^{0\dagger}\\ &= p(\psi_\mu^R\Gamma_L - \psi_\mu^L\Gamma_R)\\ &\quad -\frac{1}{\xi^2}\sum_{k\in\overline{B_0}}\sum_{\nu=1}^{2N}\psi_{k\mu}^*\psi_{k\nu}^*(\psi_{k1}^2+\psi_{k2N}^2)\gamma_\nu\\ &= -\frac{1}{\xi^2}\sum_\nu \kappa_{\mu\nu}\gamma_\nu,\end{aligned} \tag{79}$$

where $\kappa_{\mu\nu}$ are real numbers due to the fact that $\psi_{k\mu} = \psi_{k'\mu}^*$ for symmetric pairs $\omega_k^0 = -\omega_{k'}^0$ in $\overline{B_0}$. The maximum singular value of the operator in Eq. (79) is $\Lambda_\mu^{\max} = \xi^{-2}\sqrt{\sum_\nu \kappa_{\mu\nu}^2}$. This result leads us to the statement of the Lemma.

[1] O. Shtanko and R. Movassagh, Phys. Rev. Lett. **125**, 086804 (2020).
